# *Trichosporon asahii* and *Trichosporon inkin* Biofilms Produce Antifungal-Tolerant Persister Cells

**DOI:** 10.3389/fcimb.2021.645812

**Published:** 2021-04-22

**Authors:** Rossana de Aguiar Cordeiro, Ana Luiza Ribeiro Aguiar, Bruno Nascimento da Silva, Lívia Maria Galdino Pereira, Fernando Victor Monteiro Portela, Zoilo Pires de Camargo, Reginaldo Gonçalves de Lima-Neto, Débora de Souza Collares Maia Castelo-Branco, Marcos Fábio Gadelha Rocha, José Júlio Costa Sidrim

**Affiliations:** ^1^ Faculty of Medicine, Federal University of Ceará, Fortaleza, Brazil; ^2^ Department of Medicine, Discipline of Infectious Diseases, Federal University of São Paulo, São Paulo, Brazil; ^3^ Tropical Medicine Department, Federal University of Pernambuco, Recife, Brazil; ^4^ College of Veterinary, State University of Ceará, Fortaleza, Brazil

**Keywords:** antifungal susceptibility, dormant cells, *Trichosporon* spp., amphotericin B, trichosporonosis

## Abstract

Persister cells are metabolically inactive dormant cells that lie within microbial biofilms. They are phenotypic variants highly tolerant to antimicrobials and, therefore, associated with recalcitrant infections. In the present study, we investigated if *Trichosporon asahii* and *T. inkin* are able to produce persister cells. *Trichosporon* spp. are ubiquitous fungi, commonly found as commensals of the human skin and gut microbiota, and have been increasingly reported as agents of fungemia in immunocompromised patients. Biofilms derived from clinical strains of *T asahii* (n=5) and *T. inkin* (n=7) were formed in flat-bottomed microtiter plates and incubated at 35°C for 48 h, treated with 100 μg/ml amphotericin B (AMB) and incubated at 35°C for additional 24 h. Biofilms were scraped from the wells and persister cells were assayed for susceptibility to AMB. Additionally, we investigated if these persister cells were able to generate new biofilms and studied their ultrastructure and AMB susceptibility. Persister cells were detected in both *T asahii* and *T. inkin* biofilms and showed tolerance to high doses of AMB (up to 256 times higher than the minimum inhibitory concentration). Persister cells were able to generate biofilms, however they presented reduced biomass and metabolic activity, and reduced tolerance to AMB, in comparison to biofilm growth control. The present study describes the occurrence of persister cells in *Trichosporon* spp. and suggests their role in the reduced AMB susceptibility of *T*. *asahii* and *T. inkin* biofilms.

## Introduction

*Trichosporon* spp. are ubiquitous fungi, commonly found as commensals of the human skin and gastrointestinal tract (Duarte-Oliveira et al., 2017). *Trichosporon* species are emerging opportunistic fungi that have been increasingly reported as agents of fungemia in the last years, especially in immunocompromised patients (Challapilla et al., 2019; Sah et al., 2019; Alp et al., 2020). Furthermore, *Trichosporon* species have been reported as the second leading cause of invasive yeast infection in patients with malignant hematological diseases (Almeida and Hennequin, 2016).

The ability of *Trichosporon* species to cause systemic infections is likely associated with the expression of virulence factors, such as extracellular lytic compounds, and, most importantly, biofilms (Montoya et al., 2018), microbial communities surrounded by a polymeric extracellular matrix (Zhao et al., 2017). This structure expresses several mechanisms to escape the action of antimicrobials, as well as to resist physical stress, desiccation, UV radiation, and host immune system (Martinez and Casadevall, 2007; Polke et al., 2015). Previous studies have shown that *T. asahii* and *T. inkin* biofilms produce extracellular protease (Cordeiro et al., 2015) and also show increased tolerance to antifungals (Cordeiro et al., 2015; Almeida and Hennequin, 2016; Montoya et al., 2018; Cordeiro et al., 2019). Indeed, *T. asahii* biofilms may be up to 16,000 times more resistant to voriconazole, the most active antifungal against *Trichosporon* planktonic cells (Di Bonaventura et al., 2006).

Fungal biofilms tolerate high antifungal concentrations by many simultaneous mechanisms (Taff et al., 2013). Previous studies conducted with *Candida albicans* have shown that antifungal tolerance may be related to the development of a subpopulation of dormant cells within biofilms, known as persister cells (LaFleur et al., 2006). Such persister cells have been related to relapsing infections, as they tolerate antifungal exposure and can revert to an actively growing state, repopulating the biofilm, once treatment is ceased (Del Pozo, 2018).

Despite their relevance as emergent opportunistic species, few studies have addressed mechanisms of antifungal resistance of *Trichosporon* species in planktonic or sessile forms (Kushima et al., 2012; Kushima et al., 2015; Kushima et al., 2017; Li et al., 2017; Padovan et al., 2019). The present study aimed to evaluate if *T. asahii* and *T. inkin* are prone to develop dormant persister cells within biofilms and to suggest their importance in antifungal tolerance.

## Material and Methods

### Microorganisms

A total of 12 strains of *Trichosporon* spp. were evaluated in this study (**Table 1**). Strain identification was based on analysis of micromorphological features on malt agar (De Hoog et al., 2000) and sequencing of intergenic spacer region IG1 of rDNA (Silvestre Junior et al., 2010). Strains were recovered from storage and maintained on potato dextrose agar (PDA; Himedia, India), at 35°C, for 48 h. The isolates belong to the culture collection of the Specialized Medical Mycology Center of Federal University of Ceará, Brazil. The chosen *T. asahii* strains do not have mechanisms of acquired resistance to amphotericin B (wild-type), according to the epidemiological cutoff values (ECVs), proposed by Francisco et al. (2019) for the species.

**Table 1:** *Trichosporon* strains used in this study.

Species	Strain	GenBank	Source
*T. asahii*	CEMM 05-6-072	JX124945	urine
	CEMM 05-6-073	JX124961	catheter
	CEMM 03-1-072	MW291565	blood
	CEMM 80	MW291562	tracheal aspirate
	CEMM 81	MW291563	blood
*T. inkin*	CEMM 01-1-143	JX125002.1	skin lesion
	CEMM 01-1-144	JX124989.1	skin lesion
	CEMM 01-1-145	JX124958.1	urine
	CEMM 05-6-057	HM46988.1	white *piedra*
	CEMM 05-6-074	JX124953	urine
	CEMM 05-6-075	JX124985	perigenital area
	CEMM 03-1-073	MW291566	nails

*C. albicans* ATCC 10231 was included as internal control for persister cell isolation; moreover, *C. krusei* ATCC 6258 and *C. parapsilosis* ATCC 22019 were used as quality controls for susceptibility assays (CLSI, 2008).

### Drugs

Stock solution of amphotericin B (AMB; Sigma Chemical Co., St. Louis, MO, United States) was prepared as recommended by the document M27-A3 (CLSI, 2008). Serial two-fold dilutions of AMB were performed in RPMI 1640 medium (Sigma-Aldrich, MO, United States), buffered to pH 7.0 with 0.165 M morpholinepropanesulfonic acid (MOPS; Sigma-Aldrich, MO, USA).

### Biofilm Formation

Biofilm growth was induced as described by Cordeiro et al. (2015). Strains of *T. asahii* (n=5) and *T. inkin* (n=7) were grown on potato dextrose agar (PDA, Himedia, Mumbai, India) at 35°C for 48 h. Aliquots of 200 μl of fungal suspension adjusted to 2 x 10^6^ cells/ml in RPMI 1640 medium were added to flat-bottomed 96-well polystyrene microplates and incubated at 35°C for 6 h (adhesion period) at 80 rpm. After incubation, non-adherent cells were removed by washing with sterile phosphate buffer saline with 0.05% Tween 20 (PBS-Tween 20). Afterwards, the wells were filled with RPMI medium and incubated at 35°C for 48 h (maturation period) at 80 rpm.

### Detection of Persister Cells

The presence of persister cells in *Trichosporon* spp. biofilms was evaluated according to the methodology described by LaFleur et al. (2006) for *C. albicans* biofilms. Mature biofilms were washed with sterile PBS-Tween 20 and then treated with 100 μg/ml AMB and incubated at 35°C for additional 24 h (La Fleur et al., 2006). Thereafter, the wells were washed with sterile PBS-Tween 20, scraped with a pipette tip and the cells were resuspended in 100 μl in sterile PBS. Suspensions were serially diluted, plated on PDA agar and incubated at 35°C for 24 h for viable cell counting. Cells that survived exposure to 100 μg/mL AMB treatment were considered persister cells (LaFleur et al., 2006) Biofilms formed in RPMI 1640 medium without antifungal drug were included as biofilm growth control. *C. albicans* ATCC 10231 was included as control for validation of the methodology described by LaFleur et al. (2006), as it has been shown to produce dormant cells in the presence of AMB (Boucherit et al., 2007). All assays were performed in triplicate, at two independent experiments.

### Regeneration of Biofilms by Persister Cells

After observing the presence of persister cells in *Trichosporon* biofilms, we tested their ability to regenerate biofilms on an abiotic surface. Persister cells were induced and isolated as described above. Thereafter, cell suspensions were adjusted in RPMI medium, transferred to flat-bottomed 96-well polystyrene plates and incubated at 35°C for 48 h at 80 rpm order to form progeny biofilms. Results were compared with the following paired controls: (1) mature biofilms produced by planktonic cells; and (2) progeny biofilms produced by cells detached from mature biofilms. Biofilms were evaluated for metabolic activity and biomass, by the XTT reduction (Cordeiro et al., 2015) and crystal violet staining assays (Pierce et al., 2015), respectively.

### AMB Susceptibility of Persister Cells, Biofilm Cells, and Planktonic Cells

Susceptibility of persister cells to AMB was accessed by the broth microdilution method (CLSI, 2008). For isolation of persister cells, 48-h mature biofilms grown in RPMI-medium without antifungals were incubated with 100 µg/ml AMB for 24 h. Progeny biofilms were formed by inoculating persister cells into drug-free RPMI medium and repeating the biofilm growth procedure described above. Results were compared with the following paired controls: (1) planktonic cells; (2) mature biofilms produced by planktonic cells; (3) progeny biofilms produced by cells detached from mature biofilms; and (4) progeny biofilms produced by persister cells. Planktonic cells were obtained from 48-h culture on PDA at 35°C. Cell suspensions from biofilms were obtained after scraping of the wells and centrifugation at 9,167 x*g* for 10 min. Inocula were prepared in sterile saline solution and adjusted to a final concentration of 0.5–2.5 × 10^3^ cells/ml in RPMI 1640 medium buffered to pH 7.0 with 0.165 M MOPS. AMB was tested in concentrations ranging from 0.25 to 128 µg/ml (Cordeiro et al., 2015). Plates were incubated at 35°C for 48 h and fungal growth was visually determined analyzed. The minimum inhibitory concentration (MIC) was defined as the lowest concentration able of inhibiting 100% of visual fungal growth. Isolates were tested in triplicate, at two independent experiments. Controls were grown in RPMI medium without AMB. *C. krusei* ATCC 6258 and *C. parapsilosis* ATCC 22019 were included as quality controls.

### Biofilm Structure and Morphology

Structural analysis of Trichosporon biofilms (*T. asahii*, CEMM 05-6-072, urine; *T. inkin*, CEMM 05-6-074, urine) was performed by scanning electron microscopy (SEM) and Confocal Laser Scanning Microscopy (CLSM), as described elsewhere (Di Bonaventura et al., 2006; Cordeiro et al., 2017). Biofilms were formed on Thermanox^®^ slides (Thermo Fisher Scientific, NY, USA) in 12-well polystyrene plates with RPMI medium as described above and incubated at 35°C for 48 h at 80 rpm. Paired controls were grown in RPMI medium without antimicrobials. For SEM analysis, biofilms were fixed with 2.5% glutaraldehyde in 0.15M sodium cacodylate buffer and incubated overnight at 4°C. Biofilms were washed twice with 0.15M cacodylate buffer for 5 min and dehydrated in ethanol. Slides were dried with hexamethyldisilazane (Polysciences Europe, Germany) for 30 min, coated with 10nm gold (Emitech Q150T, Lewes, UK) and observed in a SEM (FEI Inspect S50, Thermo Fisher Scientific, Hillsboro, Oregon, USA) in the high vacuum mode at 15 kV. For CLSM, biofilms were stained with the Live/DeadTM (Invitrogen, Molecular Probes, Carlsbad, CA, USA) and evaluated with a confocal Nikon C2 C microscope (Nikon, Melville, NY, USA), at 488 nm, for the detection of SYTO 9 (live cells) and at 561 nm, for the detection of propidium iodide (dead/damaged cells). Images were processed using ImageJ Software (Collins, 2007). The software COMSTAT was used for quantitative analysis of the image stacks produced by CLSM.

### Statistical Analysis

Parametric data were analyzed using Student’s t-test or one-way analysis of variance (ANOVA) followed by Tukey’s post-hoc test. For data with asymmetry, Wilcoxon’s or Friedman’s nonparametric tests followed by Dunn’s post-hoc test were applied. P-values < 0.05 were considered statistically significant. Statistical Analysis was performed using the software GraphPad Prism 7.0 (GraphPad Software, CA, USA).

## Results

### Presence of Persister Cells in *Trichosporon* Biofilms

**Figure 1:**
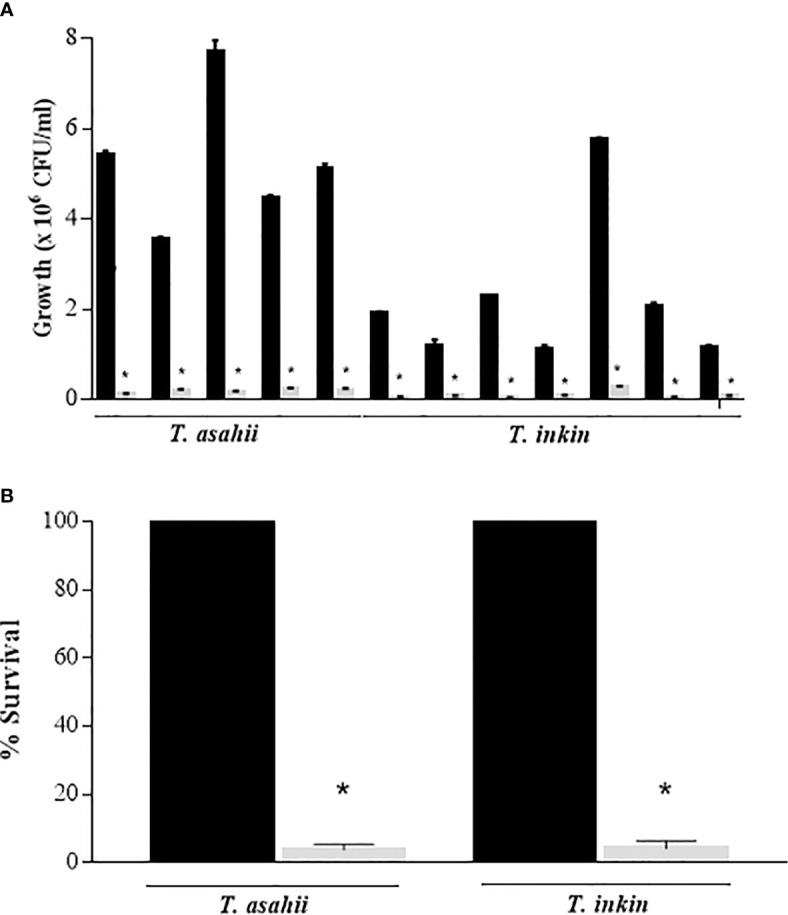
Detection of persister cells in *T. asahii* and *T. inkin* biofilms (grey bars). **(A)** Mature biofilms formed in RPMI medium were treated with 100 µg/mL AMB. **(B)** Survival of *T. asahii* and *T. inkin* biofilms challenged with 100 µg/mL AMB. Biofilm controls were grown in drug-free RPMI medium (black bars). Values are shown as mean ± SD. *Statistically significant differences when compared to the respective drug-free control (P < 0.05).

The colony-forming unit (CFU) counts showed the presence of viable cells in *Trichosporon* biofilms after exposure to 100 μg/mL AMB. Counts ranged from 1.1 x 10^5^ to 2.26 x 10^5^ CFU/mL for *T. asahii* and 2.6 x 10^4^ to 2.5 x 10^5^ *T. inkin* strains (**Figure 1A**). Biofilm growth controls ranged from 3.53 x 10^6^ to 7.7 x 10^6^ CFU/mL and 1.12 x 10^6^ to 5.75 x 10^6,^ for *T. asahii* and *T. inkin*, respectively. Persister cells consisted approximately 4% of viable cells for both *T. asahii* and *T. inkin* biofilms (**Figure 1B**). Statistically significant differences were observed when compared to drug-free growth control (P<0.05). The number of persister cells of *C. albicans* (2 x 10^4^ CFU/ml) corresponds to approximately 1.45% of the total number of cells within biofilm growth control (1.38 x 10^6^ CFU/ml).

### Persister Cells of *Trichosporon* Can Form New Biofilms

**Figure 2:**
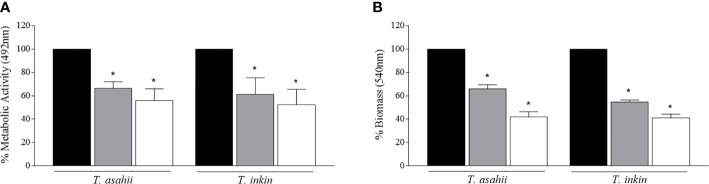
Regeneration of *T. asahii* and *T inkin* biofilms. Biofilms were formed in RPMI medium and produced by planktonic cells (black bars), cells detached from mature biofilms (grey bars) or persister cells (white bars). Metabolic activity and total biomass were expressed as relative percentages of absorbance by the XTT reduction **(A)** and crystal violet staining **(B)** assays. *Statistically significant differences when compared to the biofilm growth control produced by planktonic cells (P < 0.05).

Mature *Trichosporon* biofilms were challenged with AMB (100 µg/ml), then, progeny biofilms were formed by inoculating persister cells into RPMI medium, as previously described. Persister cells from each strain were able to form progeny biofilms. In general, a significant reduction in metabolic activity and biomass was observed for both species, when compared to progeny biofilms produced by cells detached from mature biofilms (P<0.05), as shown in **Figure 2**.

#### Persister Cells Showed Tolerance to AMB

Persister cells presented increased tolerance to AMB, compared to planktonic cells and biofilm-detached cells (**Table 2**). MICs against persister cells from *T. asahii* and *T. inkin* biofilms ranged from 64 to >128 µg/mL (geometric mean: 111.43 µg/mL and 115.93 µg/mL, respectively). MICs against planktonic cells ranged from 0.25 to 2.0 µg/mL for *T. asahii* (geometric mean: 1.41 µg/mL) and 0.5 µg/mL to 1.0 µg/mL for *T. inkin* (geometric mean: 1.0 µg/mL). MICs against mature biofilm-detached cells ranged from 16 to 64 µg/mL (geometric mean: 36.75 µg/mL and 28.98 µg/mL, for *T. asahii* and *T. inkin*, respectively). MICs against progeny biofilm-detached cells ranged from 8 to 32 µg/mL (geometric mean: 21.11 µg/mL and 14.5 µg/mL, for *T. asahii* and *T. inkin*, respectively). Cells from progeny biofilms from persister cells showed increased susceptibility to AMB, when compared to other biofilm-derived cells, displaying MICs ranging from 2 to 8 µg/mL (geometric mean: 5 µg/mL for both species). AMB MIC of control strains *C. krusei* ATCC and *C. parapsilosis* ATCC 22019 were according CLSI guidelines (CLSI, 2008).

**Table 2:** Minimum inhibitory concentration (MIC) of amphotericin B (AMB) against persister cells, biofilm cells and planktonic cells of *T. asahii* and *T. inkin* strains.

Species/Strains		AMB MIC (µg/mL)*				
		Planktonic cells^a^	Biofilm cells^b^	Progeny biofilm^c^	Persister cells^d^	Progeny biofilm-persister cells^e^
***T. asahii***	CEMM 05-6-072	0.5	32	16	128	8
	CEMM 05-6-073	1	16	16	64	8
	CEMM 03-1-072	2	32	32	128	8
	CEMM 80	0.25	64	16	>128	2
	CEMM 81	0.5	64	32	>128	4
	**Geometric mean**	1.41	36.75	21.11	111.43	5
***T. inkin***	CEMM 01-1-143	0.5	16	8	>128	8
	CEMM 01-1-144	0.5	32	16	128	4
	CEMM 01-1-145	0.5	32	16	128	4
	CEMM 05-6-057	1	32	16	128	8
	CEMM 05-6-074	0.5	16	8	64	2
	CEMM 05-6-075	1	32	16	128	8
	CEMM 03-1-073	1	64	32	128	8
	**Geometric mean**	1	28.98	14.5	115.93	5

*100% inhibition of visible fungal growth.

a Obtained from 48-h cultures on PDA at 35°C.

b Disaggregated cells from mature biofilms (48 h) produced by planktonic cells.

c Disaggregated cells from progeny mature biofilms (48 h) produced by cells detached from mature biofilms.

d Persister cells.

e Disaggregated cells from progeny mature biofilms (48 h) produced by persister cells.

#### Biofilm Ultrastructure

**Figure 3:**
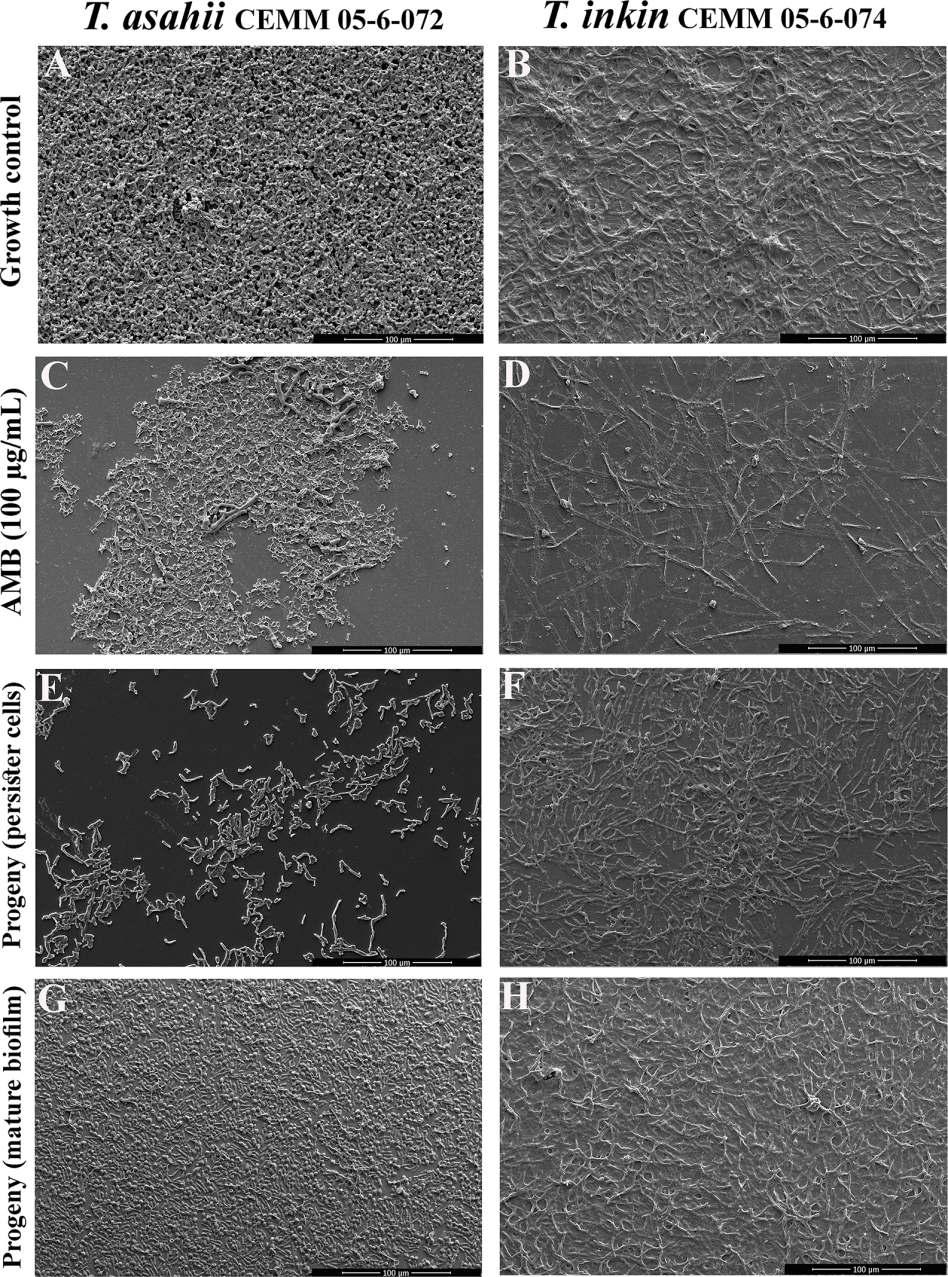
Ultrastructure of *T. asahii* CEMM 05-6-072 and *T. inkin* CEMM 05-6-074 biofilms by SEM. Sessile cells were formed on Thermanox™ coverslips in drug-free RPMI medium as controls **(A, B)**. Mature biofilms (48 h) were challenged with 100 μg/mL AMB and incubated at 35°C for 24 h **(C, D)**. Surviving cells were considered persisters. Biofilms were then scrapped with a pipette tip and inocula were prepared in RPMI medium. Progeny biofilms produced by persister cells **(E, F)** or mature biofilms-detached cells **(G, H)** were formed in RPMI medium.

**Figure 4:**
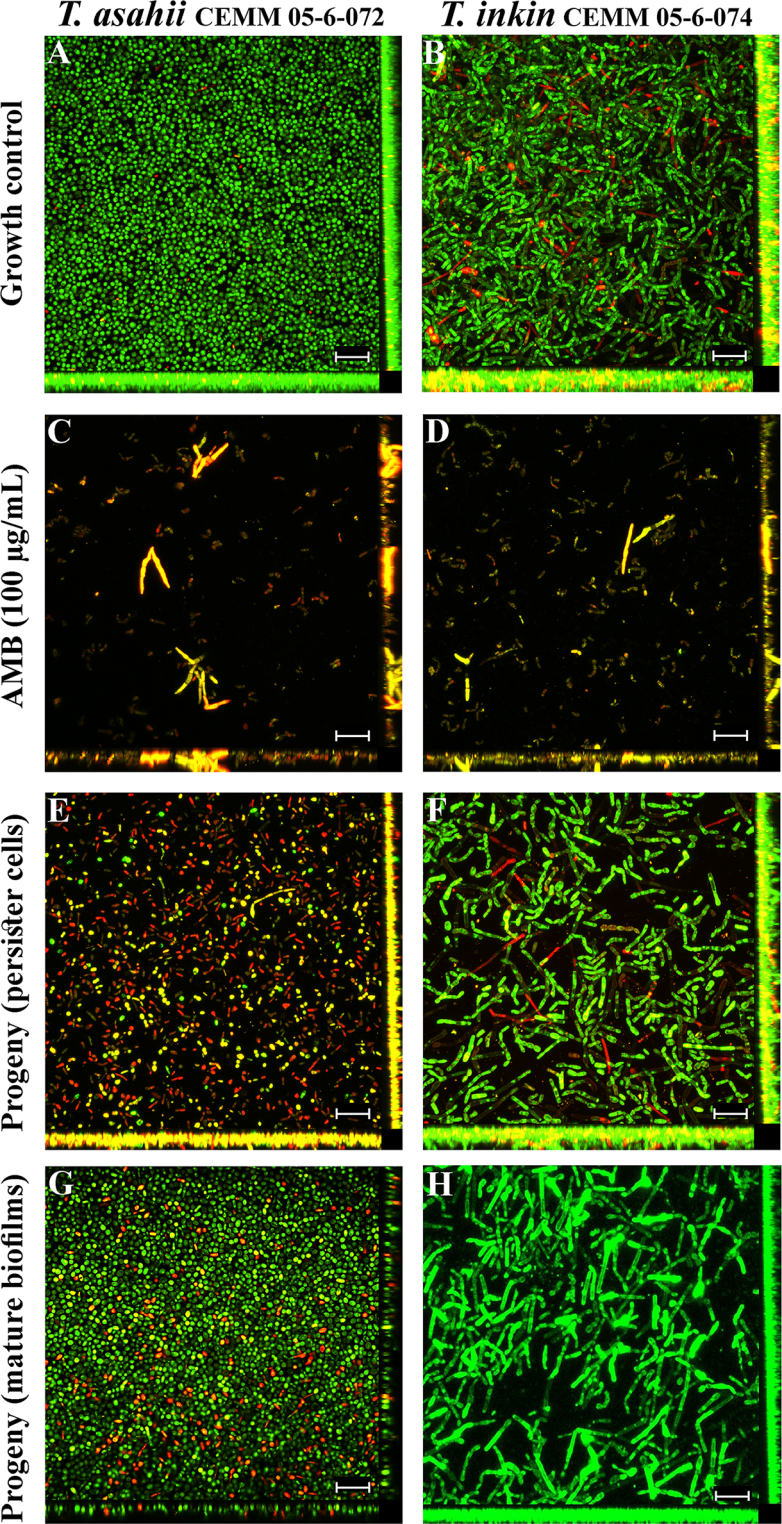
Scanning electron microscopy of *T. asahii* CEMM 05-6-072 and *T. inkin* CEMM 05-6-074 biofilms. Sessile cells were formed on Thermanox™ coverslips in drug-free RPMI medium as controls **(A, B)**. Mature biofilms (48 h) were challenged with 100 μg/mL AMB **(C, D)** for detection of persister cells. Progeny biofilms produced by persister cells **(E, F)** or mature biofilms-detached cells were formed in RPMI medium **(G, H)**. Live cells are shown in green and dead/damaged cells are shown in yellow/red after stain with SYTO9 and propidium iodide, respectively. Magnification: 400x. Bar: 100 μm.

Persister cells were compared to biofilm growth control by SEM (**Figure 3**) and CLSM (**Figure 4**). Structured biofilms with high cell density enclosed by a dense extracellular material were seen in drug-free growth controls (**Figures 3A, B**, and **4A, B**). AMB (100 µg/mL) was able to disrupt the three-dimensional structure of mature biofilms, leaving deformed fungal structures (**Figures 3C, D**) and rare live cells (**Figures 4C, D**). Persister cells were able to form progeny biofilms with fewer filaments and less extracellular matrix **(Figures 3E, F** and **4E, F**) than biofilm growth control. Progeny biofilms produced by cells detached from mature biofilms also showed fewer filaments and less extracellular matrix than the drug-free growth control (**Figures 3G, H** and **4G, H**).

## Discussion

Mechanisms associated with antifungal tolerance have already been described in *Candida* biofilms and include increased efflux pump activity; drug sequestration by extracellular matrix (Cohen et al., 2013; Wuyts et al., 2018); expression of resistance genes (Li et al., 2015); reduction of cell ergosterol concentration (Mukherjee et al., 2003) and production of persistent cells (LaFleur et al., 2006; Al-Dhaheri and Douglas, 2008; Delarze and Sanglard, 2015; Wuyts et al., 2018). Although the antifungal tolerance in *Trichosporon* biofilms is widely reported (Falk et al., 2003; Di Bonaventura et al., 2006; Iturrieta-González et al., 2014; Cordeiro et al., 2015), the mechanisms underlying this phenotype have not been described.

LaFleur et al. (2006) were the first to describe the occurrence of persister cells in fungal biofilms, presenting evidence that this phenomenon occurs in *C. albicans* biofilms. Authors challenged mature biofilms with up to 900 µg/mL of AMB and showed that most of the population was killed by AMB at low concentrations but nearly 1% of the cells seemed to be completely invulnerable to higher concentrations of the drug. Since then, persister cells have been described in biofilms of *C. parapsilosis*, *C. krusei* (Al-Dhaheri and Douglas, 2008) and *Saccharomyces cerevisiae* (Bojsen et al., 2014).

Persister cells are phenotypic variants that are maintained in a state of dormancy, being able to survive the oxidative stress induced by high concentrations of antifungals through the activation of energy storage pathways (Wuyts et al., 2018). As these dormant cells have low metabolic activity, they have been detected by counting colony-forming units, instead of using viability reagents (LaFleur et al., 2006; Al-Dhaheri and Douglas, 2008; LaFleur et al., 2010; Li et al., 2015; Sun et al., 2016; Wu et al., 2019; Yee et al., 2019; Galdiero et al., 2020; Wu et al., 2020). In the present study, we observed that clinical strains of *T. asahii* and *T. inkin* produced persister cells inside their biofilms. Using the methodology originally proposed by LaFleur for the detection of persister cells in *C. albicans* biofilms, it was observed that the proportion of persister cells varied from 2 to 5% for *T. asahii* and from 2 to 6% for *T. inkin*. These values are higher than those found in *C. albicans* (LaFleur et al., 2006), *C. parapsilosis* and *C. krusei* biofilms (Al-Dhaheri and Douglas, 2008), which usually comprise up to 2% of the total population. We do not know if these results indicate intrinsic properties of *Trichosporon* biofilms or if they are derived from the small number of strains investigated in the present study.

The concept of persister cells also encompass a biphasic pattern of killing when a sessile population is challenged with an antifungal drug at concentrations well above MIC (Wuyts et al., 2018). In our study, mature biofilms of *Trichosporon* were challenged with increased concentrations of AMB (10, 50 and 100 µg/mL) and a biphasic pattern of killing was seen: whereas 50 µg/mL AMB suppressed *Trichosporon* biofilms (data not shown), few cells remained alive after challenge with 100 µg/mL of AMB, as shown by CFU counting and confocal microscopy. Previous studies have shown that MIC values for AMB against *Trichosporon* biofilm-cells were, in general, below 50 µg/mL (Iturrieta-González et al., 2014; Cordeiro et al., 2015; Montoya et al., 2018).

In the present study, persister cells of *T. asahii* and *T. inkin* were able to originate progeny biofilms, however, these biofilms presented less biomass and reduced metabolic activity when compared to the progeny biofilms produced by cells detached from mature biofilms. Such results were corroborated by the ultrastructural analysis, which revealed that the biofilms deriving from persister cells have lower cell density. Detached cells from these progeny biofilms were less tolerant to AMB than persister cells contained in the starter inoculum. These results suggest that *Trichosporon* persister cells are not mutants but phenotypic variants of regular cells, as previously demonstrated for other fungal species (LaFleur et al., 2006; Al-Dhaheri and Douglas, 2008; Wuyts et al., 2018).

Besides the intracellular accumulation of energy storage molecules (Li et al., 2015; Wuyts et al., 2018), it is supposed that persister cells may have a different cell wall composition (Wuyts et al., 2018) and increased extracellular matrix production (Li et al., 2015). Previous studies have shown that the extracellular matrix actively contributes to the antifungal resistance of biofilms, by inducing drug-sequestration and harboring drug-efflux proteins (Li et al., 2015; Wuyts et al., 2018; Berman and Krysan, 2020). Although we were not able to study the cell wall properties of persister cells of *T. asahii* and *T. inkin*, microscopy analyses showed that these cells and their progeny biofilms had less extracellular matrix, resulting in thinner biofilms. The reduced amount of extracellular matrix observed in our results reinforces the hypothesis that the tolerance of *Trichosporon* biofilms to AMB at 100 µg/mL is derived from the presence of persister cells.

Experimental evidences suggest that persister cells are directly related to the occurrence of recalcitrant microbial infections. Unequivocal proof was first presented by Mulcahy et al. (2010) who showed a direct relationship between the presence of persister cells of *Pseudomonas aeruginosa* in patients with cystic fibrosis pneumonia. Studies performed with persister cells from bacterial biofilms prove that these cells, besides presenting reduced susceptibility to antibiotics, are better suited to survive the host immune system, persist within catheter-associated biofilm infection (Daubert et al., 2020) and cause more severe disease (Yee et al., 2019). Regarding fungal infections, LaFleur et al. (2010) showed that *in vivo* selection for high-persister mutants occurs in cancer patients that harbor *Candida*. Recently, it was hypothesized that persister cells inside biotic biofilms formed on vaginal epithelium and underlying tissues are related to recalcitrant vulvovaginal candidiasis (Wu et al., 2019; Wu et al., 2020). Although the virulence of fungal persister cells is not fully understood, the occurrence of dormant cells in biotic biofilms demands the attention of clinicians and researchers, since the available antifungals have low activity against biofilms and persister cells (Galdiero et al., 2020).

In conclusion, the results presented here show that *T. asahii* and *T. inkin* produce persister cells in their biofilms. These cells have a high tolerance to AMB and provide an inoculum for cell growth and new biofilm formation. Future studies should be conducted in order to understand the genetic regulation associated with the production of persister cells in *Trichosporon* biofilms and their phenotypic tolerance to AMB. Physicians should be aware that the production of persister cells within biofilms may be related to therapeutic failure in invasive trichosporonosis.

## Data Availability Statement

The original contributions presented in the study are included in the article/supplementary material. Further inquiries can be directed to the corresponding author.

## Author Contributions

RC and AA designed the research, analyzed the data and wrote the manuscript. AA, BS, LP, and FP performed the experiments. DC-B captured CLSM images. DC-B, ZC, RL-N, MR, and JC critically revised the manuscript. All authors contributed to the article and approved the submitted version.

## Conflict of Interest

The authors declare that the research was conducted in the absence of any commercial or financial relationships that could be construed as a potential conflict of interest.
